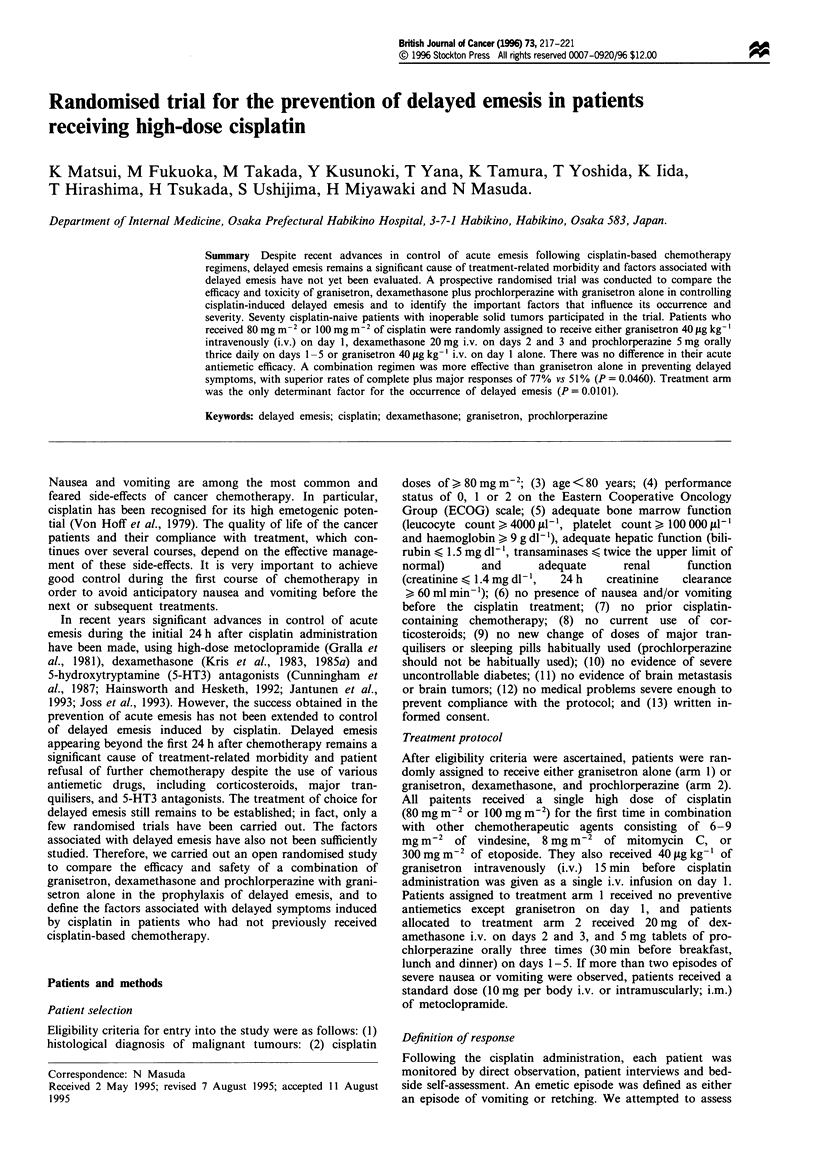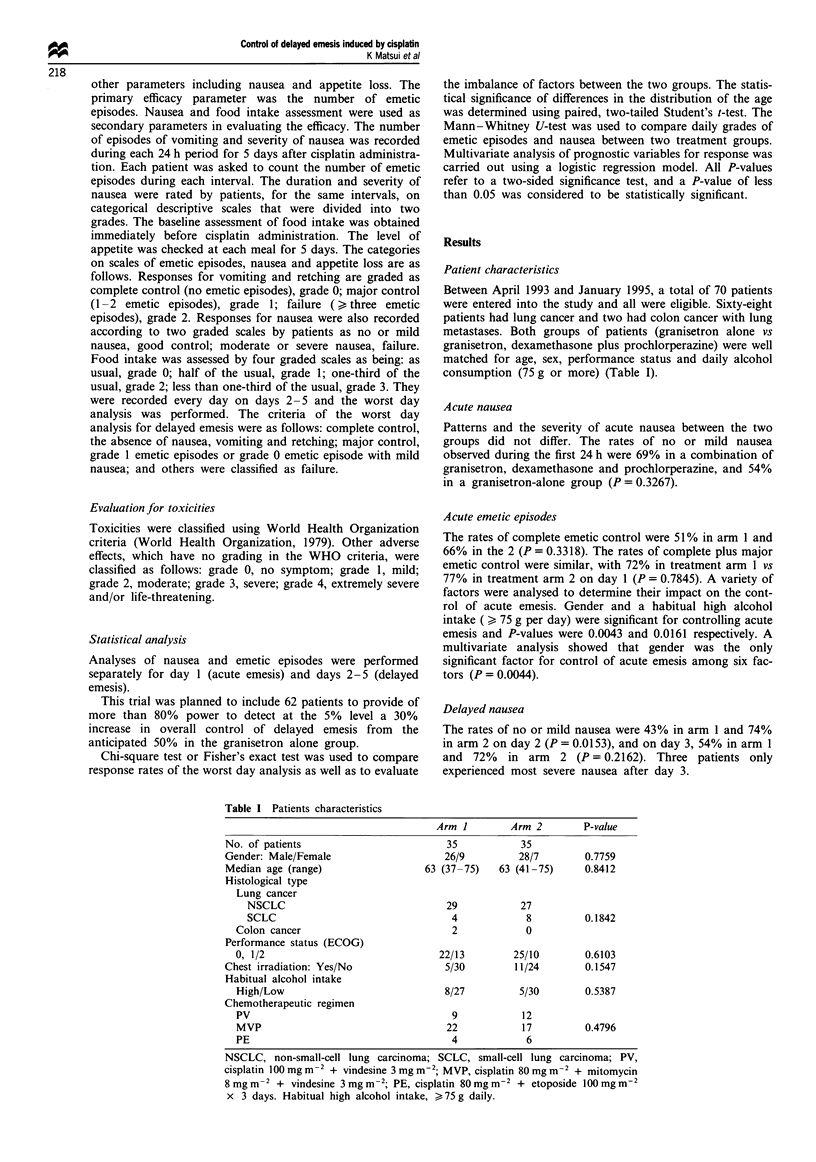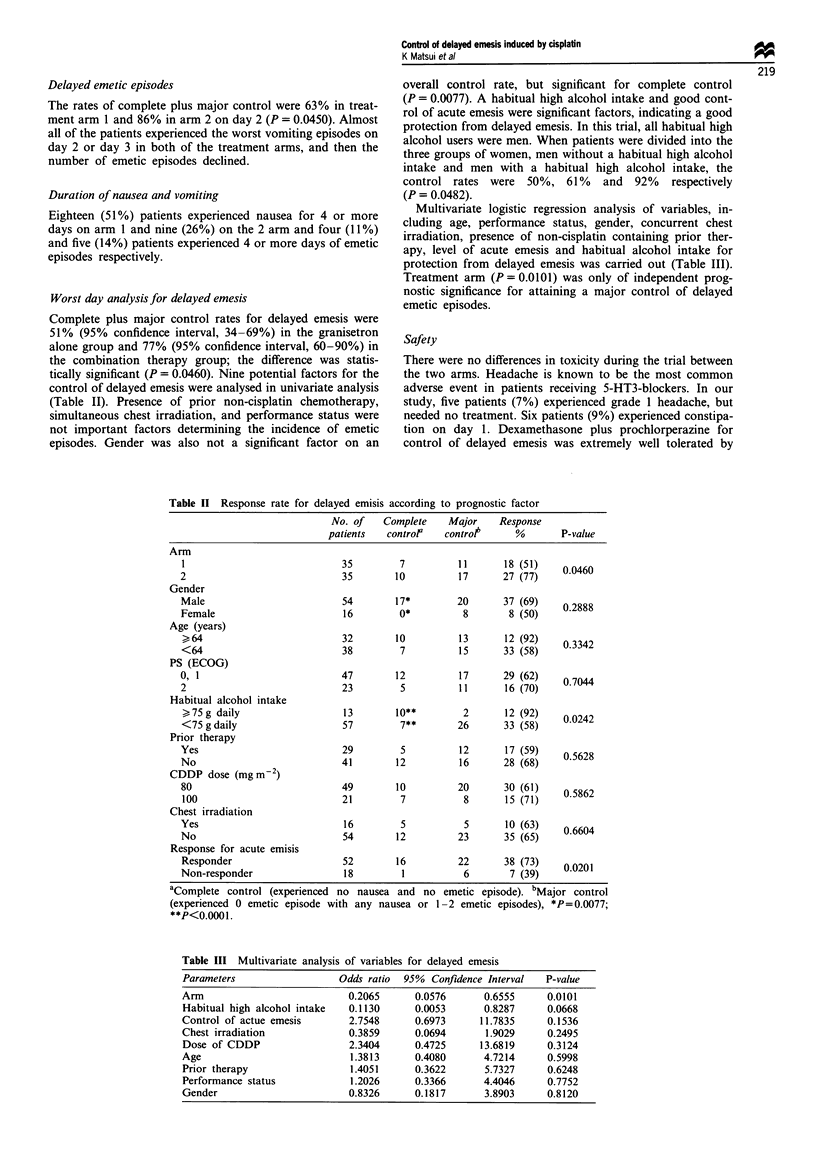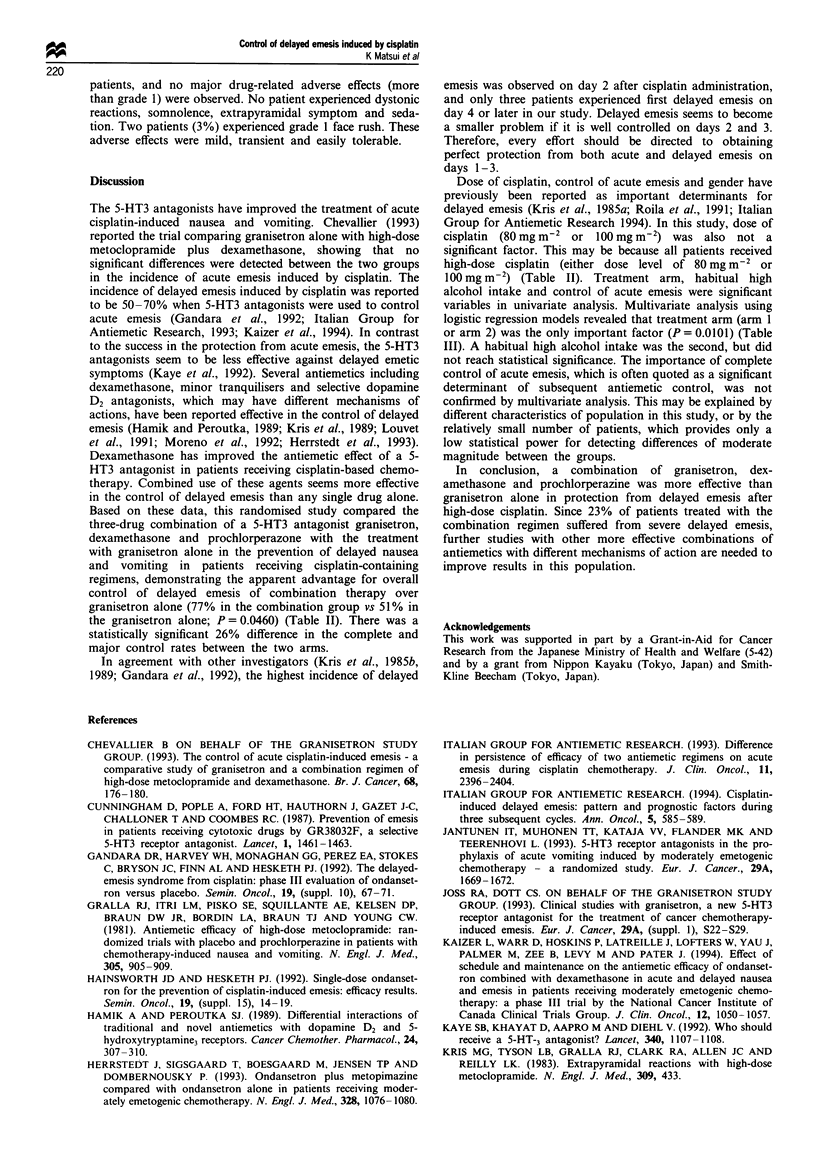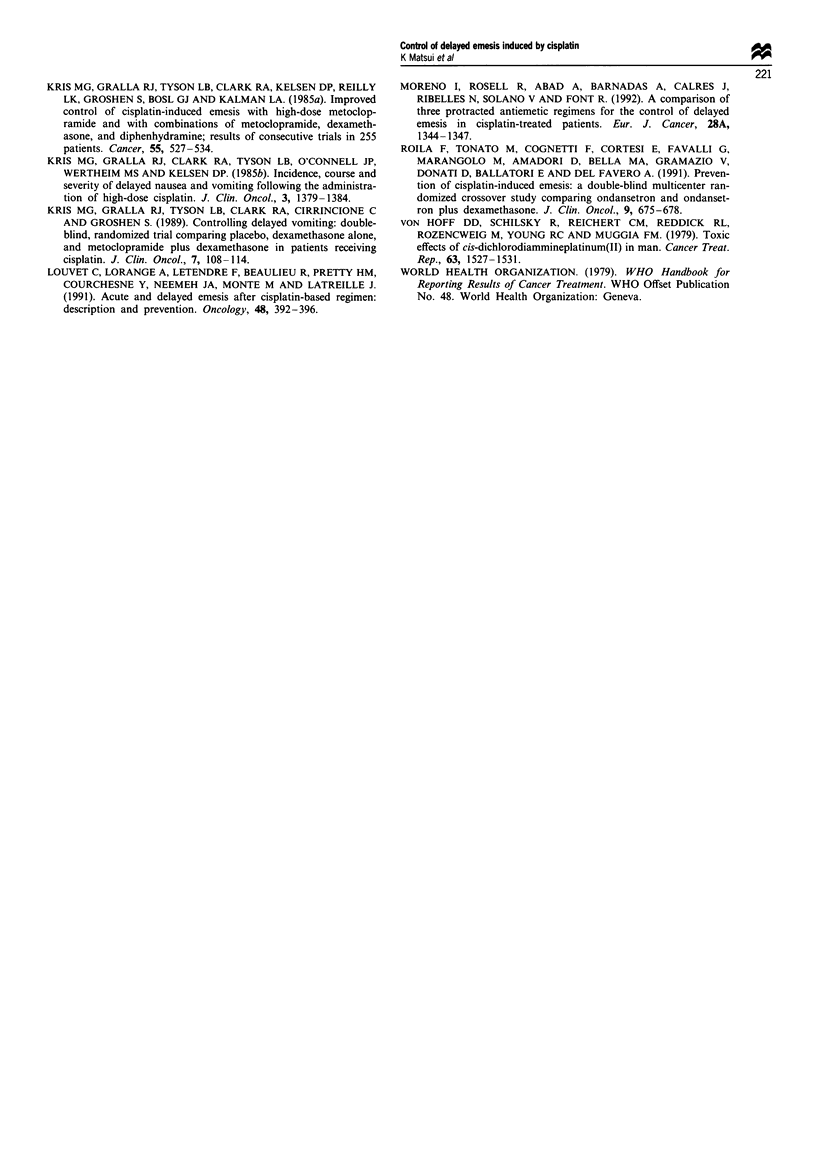# Randomised trial for the prevention of delayed emesis in patients receiving high-dose cisplatin.

**DOI:** 10.1038/bjc.1996.38

**Published:** 1996-01

**Authors:** K. Matsui, M. Fukuoka, M. Takada, Y. Kusunoki, T. Yana, K. Tamura, T. Yoshida, K. Iida, T. Hirashima, H. Tsukada, S. Ushijima, H. Miyawaki, N. Masuda

**Affiliations:** Department of Internal Medicine, Osaka Prefectural Habikino Hospital, Japan.

## Abstract

Despite recent advances in control of acute emesis following cisplatin-based chemotherapy regimens, delayed emesis remains a significant cause of treatment-related morbidity and factors associated with delayed emesis have not yet been evaluated. A prospective randomised trial was conducted to compare the efficacy and toxicity of granisetron, dexamethasone plus prochlorperazine with granisetron alone in controlling cisplatin-induced delayed emesis and to identify the important factors that influence its occurrence and severity. Seventy cisplatin-naive patients with inoperable solid tumors participated in the trial. Patients who received 80 mg m-2 or 100 mg m-2 of cisplatin were randomly assigned to receive either granisetron 40 micrograms kg-1 intravenously (i.v.) on day 1, dexamethasone 20 mg i.v. on days 2 and 3 and prochlorperazine 5 mg orally thrice daily on days 1-5 or granisetron 40 micrograms kg-1 i.v. on day 1 alone. There was no difference in their acute antiemetic efficacy. A combination regimen was more effective than granisetron alone in preventing delayed symptoms, with superior rates of complete plus major responses of 77% vs 51% (P = 0.0460). Treatment arm was the only determinant factor for the occurrence of delayed emesis (P = 0.0101).


British Journal of Cancer (1996) 73, 217–221
© 1996 Stockton Press All rights reserved 0007-0920/96 $12.00

### Randomised trial for the prevention of delayed emesis in patients receiving high-dose cisplatin

K Matsui, M Fukuoka, M Takada, Y Kusunoki, T Yana, K Tamura, T Yoshida, K Iida, T Hirashima, H Tsukada, S Ushijima, H Miyawaki and N Masuda.

*Department of Internal Medicine, Osaka Prefectural Habikino Hospital, 3-7-1 Habikino, Habikino, Osaka 583, Japan.*

**Summary** Despite recent advances in control of acute emesis following cisplatin-based chemotherapy regimens, delayed emesis remains a significant cause of treatment-related morbidity and factors associated with delayed emesis have not yet been evaluated. A prospective randomised trial was conducted to compare the efficacy and toxicity of granisetron, dexamethasone plus prochlorperazine with granisetron alone in controlling cisplatin-induced delayed emesis and to identify the important factors that influence its occurrence and severity. Seventy cisplatin-naive patients with inoperable solid tumors participated in the trial. Patients who received 80 mg $m^{-2}$ or 100 mg $m^{-2}$ of cisplatin were randomly assigned to receive either granisetron 40 $\mu$g $kg^{-1}$ intravenously (i.v.) on day 1, dexamethasone 20 mg i.v. on days 2 and 3 and prochlorperazine 5 mg orally thrice daily on days 1–5 or granisetron 40 $\mu$g $kg^{-1}$ i.v. on day 1 alone. There was no difference in their acute antiemetic efficacy. A combination regimen was more effective than granisetron alone in preventing delayed symptoms, with superior rates of complete plus major responses of 77% *vs* 51% ($P = 0.0460$). Treatment arm was the only determinant factor for the occurrence of delayed emesis ($P = 0.0101$).

**Keywords:** delayed emesis; cisplatin; dexamethasone; granisetron, prochlorperazine

Nausea and vomiting are among the most common and feared side-effects of cancer chemotherapy. In particular, cisplatin has been recognised for its high emetogenic potential (Von Hoff *et al.*, 1979). The quality of life of the cancer patients and their compliance with treatment, which continues over several courses, depend on the effective management of these side-effects. It is very important to achieve good control during the first course of chemotherapy in order to avoid anticipatory nausea and vomiting before the next or subsequent treatments.

In recent years significant advances in control of acute emesis during the initial 24 h after cisplatin administration have been made, using high-dose metoclopramide (Gralla *et al.*, 1981), dexamethasone (Kris *et al.*, 1983, 1985*a*) and 5-hydroxytryptamine (5-HT3) antagonists (Cunningham *et al.*, 1987; Hainsworth and Hesketh, 1992; Jantunen *et al.*, 1993; Joss *et al.*, 1993). However, the success obtained in the prevention of acute emesis has not been extended to control of delayed emesis induced by cisplatin. Delayed emesis appearing beyond the first 24 h after chemotherapy remains a significant cause of treatment-related morbidity and patient refusal of further chemotherapy despite the use of various antiemetic drugs, including corticosteroids, major tranquilisers, and 5-HT3 antagonists. The treatment of choice for delayed emesis still remains to be established; in fact, only a few randomised trials have been carried out. The factors associated with delayed emesis have also not been sufficiently studied. Therefore, we carried out an open randomised study to compare the efficacy and safety of a combination of granisetron, dexamethasone and prochlorperazine with granisetron alone in the prophylaxis of delayed emesis, and to define the factors associated with delayed symptoms induced by cisplatin in patients who had not previously received cisplatin-based chemotherapy.

### Patients and methods

### *Patient selection*

Eligibility criteria for entry into the study were as follows: (1) histological diagnosis of malignant tumours: (2) cisplatin doses of $\geqslant 80$ mg $m^{-2}$; (3) age $<80$ years; (4) performance status of 0, 1 or 2 on the Eastern Cooperative Oncology Group (ECOG) scale; (5) adequate bone marrow function (leucocyte count $\geqslant 4000$ $\mu l^{-1}$, platelet count $\geqslant 100\,000$ $\mu l^{-1}$ and haemoglobin $\geqslant 9$ g $dl^{-1}$), adequate hepatic function (bilirubin $\leqslant 1.5$ mg $dl^{-1}$, transaminases $\leqslant$ twice the upper limit of normal) and adequate renal function (creatinine $\leqslant 1.4$ mg $dl^{-1}$, 24 h creatinine clearance $\geqslant 60$ ml $min^{-1}$); (6) no presence of nausea and/or vomiting before the cisplatin treatment; (7) no prior cisplatin-containing chemotherapy; (8) no current use of corticosteroids; (9) no new change of doses of major tranquilisers or sleeping pills habitually used (prochlorperazine should not be habitually used); (10) no evidence of severe uncontrollable diabetes; (11) no evidence of brain metastasis or brain tumors; (12) no medical problems severe enough to prevent compliance with the protocol; and (13) written informed consent.

### *Treatment protocol*

After eligibility criteria were ascertained, patients were randomly assigned to receive either granisetron alone (arm 1) or granisetron, dexamethasone, and prochlorperazine (arm 2). All paitents received a single high dose of cisplatin (80 mg $m^{-2}$ or 100 mg $m^{-2}$) for the first time in combination with other chemotherapeutic agents consisting of 6–9 mg $m^{-2}$ of vindesine, 8 mg $m^{-2}$ of mitomycin C, or 300 mg $m^{-2}$ of etoposide. They also received 40 $\mu$g $kg^{-1}$ of granisetron intravenously (i.v.) 15 min before cisplatin administration was given as a single i.v. infusion on day 1. Patients assigned to treatment arm 1 received no preventive antiemetics except granisetron on day 1, and patients allocated to treatment arm 2 received 20 mg of dexamethasone i.v. on days 2 and 3, and 5 mg tablets of prochlorperazine orally three times (30 min before breakfast, lunch and dinner) on days 1–5. If more than two episodes of severe nausea or vomiting were observed, patients received a standard dose (10 mg per body i.v. or intramuscularly; i.m.) of metoclopramide.

### *Definition of response*

Following the cisplatin administration, each patient was monitored by direct observation, patient interviews and bedside self-assessment. An emetic episode was defined as either an episode of vomiting or retching. We attempted to assess

Correspondence: N Masuda
Received 2 May 1995; revised 7 August 1995; accepted 11 August 1995

other parameters including nausea and appetite loss. The primary efficacy parameter was the number of emetic episodes. Nausea and food intake assessment were used as secondary parameters in evaluating the efficacy. The number of episodes of vomiting and severity of nausea was recorded during each 24 h period for 5 days after cisplatin administration. Each patient was asked to count the number of emetic episodes during each interval. The duration and severity of nausea were rated by patients, for the same intervals, on categorical descriptive scales that were divided into two grades. The baseline assessment of food intake was obtained immediately before cisplatin administration. The level of appetite was checked at each meal for 5 days. The categories on scales of emetic episodes, nausea and appetite loss are as follows. Responses for vomiting and retching are graded as complete control (no emetic episodes), grade 0; major control (1–2 emetic episodes), grade 1; failure ($\geqslant$ three emetic episodes), grade 2. Responses for nausea were also recorded according to two graded scales by patients as no or mild nausea, good control; moderate or severe nausea, failure. Food intake was assessed by four graded scales as being: as usual, grade 0; half of the usual, grade 1; one-third of the usual, grade 2; less than one-third of the usual, grade 3. They were recorded every day on days 2–5 and the worst day analysis was performed. The criteria of the worst day analysis for delayed emesis were as follows: complete control, the absence of nausea, vomiting and retching; major control, grade 1 emetic episodes or grade 0 emetic episode with mild nausea; and others were classified as failure.

### *Evaluation for toxicities*

Toxicities were classified using World Health Organization criteria (World Health Organization, 1979). Other adverse effects, which have no grading in the WHO criteria, were classified as follows: grade 0, no symptom; grade 1, mild; grade 2, moderate; grade 3, severe; grade 4, extremely severe and/or life-threatening.

### *Statistical analysis*

Analyses of nausea and emetic episodes were performed separately for day 1 (acute emesis) and days 2–5 (delayed emesis).

This trial was planned to include 62 patients to provide of more than 80% power to detect at the 5% level a 30% increase in overall control of delayed emesis from the anticipated 50% in the granisetron alone group.

Chi-square test or Fisher's exact test was used to compare response rates of the worst day analysis as well as to evaluate the imbalance of factors between the two groups. The statistical significance of differences in the distribution of the age was determined using paired, two-tailed Student's *t*-test. The Mann–Whitney *U*-test was used to compare daily grades of emetic episodes and nausea between two treatment groups. Multivariate analysis of prognostic variables for response was carried out using a logistic regression model. All *P*-values refer to a two-sided significance test, and a *P*-value of less than 0.05 was considered to be statistically significant.

### Results

### *Patient characteristics*

Between April 1993 and January 1995, a total of 70 patients were entered into the study and all were eligible. Sixty-eight patients had lung cancer and two had colon cancer with lung metastases. Both groups of patients (granisetron alone *vs* granisetron, dexamethasone plus prochlorperazine) were well matched for age, sex, performance status and daily alcohol consumption (75 g or more) (Table I).

### *Acute nausea*

Patterns and the severity of acute nausea between the two groups did not differ. The rates of no or mild nausea observed during the first 24 h were 69% in a combination of granisetron, dexamethasone and prochlorperazine, and 54% in a granisetron-alone group ($P = 0.3267$).

### *Acute emetic episodes*

The rates of complete emetic control were 51% in arm 1 and 66% in the 2 ($P = 0.3318$). The rates of complete plus major emetic control were similar, with 72% in treatment arm 1 *vs* 77% in treatment arm 2 on day 1 ($P = 0.7845$). A variety of factors were analysed to determine their impact on the control of acute emesis. Gender and a habitual high alcohol intake ($\geqslant$ 75 g per day) were significant for controlling acute emesis and *P*-values were 0.0043 and 0.0161 respectively. A multivariate analysis showed that gender was the only significant factor for control of acute emesis among six factors ($P = 0.0044$).

### *Delayed nausea*

The rates of no or mild nausea were 43% in arm 1 and 74% in arm 2 on day 2 ($P = 0.0153$), and on day 3, 54% in arm 1 and 72% in arm 2 ($P = 0.2162$). Three patients only experienced most severe nausea after day 3.

**Table I** Patients characteristics

| | *Arm 1* | *Arm 2* | P-*value* |
|---|---|---|---|
| No. of patients | 35 | 35 | |
| Gender: Male/Female | 26/9 | 28/7 | 0.7759 |
| Median age (range) | 63 (37–75) | 63 (41–75) | 0.8412 |
| Histological type | | | |
| Lung cancer | | | |
| NSCLC | 29 | 27 | |
| SCLC | 4 | 8 | 0.1842 |
| Colon cancer | 2 | 0 | |
| Performance status (ECOG) | | | |
| 0, 1/2 | 22/13 | 25/10 | 0.6103 |
| Chest irradiation: Yes/No | 5/30 | 11/24 | 0.1547 |
| Habitual alcohol intake | | | |
| High/Low | 8/27 | 5/30 | 0.5387 |
| Chemotherapeutic regimen | | | |
| PV | 9 | 12 | |
| MVP | 22 | 17 | 0.4796 |
| PE | 4 | 6 | |

NSCLC, non-small-cell lung carcinoma; SCLC, small-cell lung carcinoma; PV, cisplatin 100 mg m$^{-2}$ + vindesine 3 mg m$^{-2}$; MVP, cisplatin 80 mg m$^{-2}$ + mitomycin 8 mg m$^{-2}$ + vindesine 3 mg m$^{-2}$; PE, cisplatin 80 mg m$^{-2}$ + etoposide 100 mg m$^{-2}$ × 3 days. Habitual high alcohol intake, $\geqslant$75 g daily.

*Delayed emetic episodes*

The rates of complete plus major control were 63% in treatment arm 1 and 86% in arm 2 on day 2 ($P = 0.0450$). Almost all of the patients experienced the worst vomiting episodes on day 2 or day 3 in both of the treatment arms, and then the number of emetic episodes declined.

*Duration of nausea and vomiting*

Eighteen (51%) patients experienced nausea for 4 or more days on arm 1 and nine (26%) on the 2 arm and four (11%) and five (14%) patients experienced 4 or more days of emetic episodes respectively.

*Worst day analysis for delayed emesis*

Complete plus major control rates for delayed emesis were 51% (95% confidence interval, 34–69%) in the granisetron alone group and 77% (95% confidence interval, 60–90%) in the combination therapy group; the difference was statistically significant ($P = 0.0460$). Nine potential factors for the control of delayed emesis were analysed in univariate analysis (Table II). Presence of prior non-cisplatin chemotherapy, simultaneous chest irradiation, and performance status were not important factors determining the incidence of emetic episodes. Gender was also not a significant factor on an overall control rate, but significant for complete control ($P = 0.0077$). A habitual high alcohol intake and good control of acute emesis were significant factors, indicating a good protection from delayed emesis. In this trial, all habitual high alcohol users were men. When patients were divided into the three groups of women, men without a habitual high alcohol intake and men with a habitual high alcohol intake, the control rates were 50%, 61% and 92% respectively ($P = 0.0482$).

Multivariate logistic regression analysis of variables, including age, performance status, gender, concurrent chest irradiation, presence of non-cisplatin containing prior therapy, level of acute emesis and habitual alcohol intake for protection from delayed emesis was carried out (Table III). Treatment arm ($P = 0.0101$) was only of independent prognostic significance for attaining a major control of delayed emetic episodes.

*Safety*

There were no differences in toxicity during the trial between the two arms. Headache is known to be the most common adverse event in patients receiving 5-HT3-blockers. In our study, five patients (7%) experienced grade 1 headache, but needed no treatment. Six patients (9%) experienced constipation on day 1. Dexamethasone plus prochlorperazine for control of delayed emesis was extremely well tolerated by

**Table II** Response rate for delayed emisis according to prognostic factor

| | *No. of patients* | *Complete control*[a] | *Major control*[b] | *Response %* | P-*value* |
|---|---|---|---|---|---|
| Arm | | | | | |
| 1 | 35 | 7 | 11 | 18 (51) | 0.0460 |
| 2 | 35 | 10 | 17 | 27 (77) | |
| Gender | | | | | |
| Male | 54 | 17* | 20 | 37 (69) | 0.2888 |
| Female | 16 | 0* | 8 | 8 (50) | |
| Age (years) | | | | | |
| ⩾64 | 32 | 10 | 13 | 12 (92) | 0.3342 |
| <64 | 38 | 7 | 15 | 33 (58) | |
| PS (ECOG) | | | | | |
| 0, 1 | 47 | 12 | 17 | 29 (62) | 0.7044 |
| 2 | 23 | 5 | 11 | 16 (70) | |
| Habitual alcohol intake | | | | | |
| ⩾75 g daily | 13 | 10** | 2 | 12 (92) | 0.0242 |
| <75 g daily | 57 | 7** | 26 | 33 (58) | |
| Prior therapy | | | | | |
| Yes | 29 | 5 | 12 | 17 (59) | 0.5628 |
| No | 41 | 12 | 16 | 28 (68) | |
| CDDP dose (mg m$^{-2}$) | | | | | |
| 80 | 49 | 10 | 20 | 30 (61) | 0.5862 |
| 100 | 21 | 7 | 8 | 15 (71) | |
| Chest irradiation | | | | | |
| Yes | 16 | 5 | 5 | 10 (63) | 0.6604 |
| No | 54 | 12 | 23 | 35 (65) | |
| Response for acute emisis | | | | | |
| Responder | 52 | 16 | 22 | 38 (73) | 0.0201 |
| Non-responder | 18 | 1 | 6 | 7 (39) | |

[a]Complete control (experienced no nausea and no emetic episode). [b]Major control (experienced 0 emetic episode with any nausea or 1–2 emetic episodes), $^{*}P=0.0077$; $^{**}P<0.0001$.

**Table III** Multivariate analysis of variables for delayed emesis

| *Parameters* | *Odds ratio* | *95% Confidence Interval* | | P-*value* |
|---|---|---|---|---|
| Arm | 0.2065 | 0.0576 | 0.6555 | 0.0101 |
| Habitual high alcohol intake | 0.1130 | 0.0053 | 0.8287 | 0.0668 |
| Control of actue emesis | 2.7548 | 0.6973 | 11.7835 | 0.1536 |
| Chest irradiation | 0.3859 | 0.0694 | 1.9029 | 0.2495 |
| Dose of CDDP | 2.3404 | 0.4725 | 13.6819 | 0.3124 |
| Age | 1.3813 | 0.4080 | 4.7214 | 0.5998 |
| Prior therapy | 1.4051 | 0.3622 | 5.7327 | 0.6248 |
| Performance status | 1.2026 | 0.3366 | 4.4046 | 0.7752 |
| Gender | 0.8326 | 0.1817 | 3.8903 | 0.8120 |

patients, and no major drug-related adverse effects (more than grade 1) were observed. No patient experienced dystonic reactions, somnolence, extrapyramidal symptom and sedation. Two patients (3%) experienced grade 1 face rush. These adverse effects were mild, transient and easily tolerable.

### Discussion

The 5-HT3 antagonists have improved the treatment of acute cisplatin-induced nausea and vomiting. Chevallier (1993) reported the trial comparing granisetron alone with high-dose metoclopramide plus dexamethasone, showing that no significant differences were detected between the two groups in the incidence of acute emesis induced by cisplatin. The incidence of delayed emesis induced by cisplatin was reported to be 50–70% when 5-HT3 antagonists were used to control acute emesis (Gandara *et al.*, 1992; Italian Group for Antiemetic Research, 1993; Kaizer *et al.*, 1994). In contrast to the success in the protection from acute emesis, the 5-HT3 antagonists seem to be less effective against delayed emetic symptoms (Kaye *et al.*, 1992). Several antiemetics including dexamethasone, minor tranquilisers and selective dopamine $D_2$ antagonists, which may have different mechanisms of actions, have been reported effective in the control of delayed emesis (Hamik and Peroutka, 1989; Kris *et al.*, 1989; Louvet *et al.*, 1991; Moreno *et al.*, 1992; Herrstedt *et al.*, 1993). Dexamethasone has improved the antiemetic effect of a 5-HT3 antagonist in patients receiving cisplatin-based chemotherapy. Combined use of these agents seems more effective in the control of delayed emesis than any single drug alone. Based on these data, this randomised study compared the three-drug combination of a 5-HT3 antagonist granisetron, dexamethasone and prochlorperazone with the treatment with granisetron alone in the prevention of delayed nausea and vomiting in patients receiving cisplatin-containing regimens, demonstrating the apparent advantage for overall control of delayed emesis of combination therapy over granisetron alone (77% in the combination group *vs* 51% in the granisetron alone; $P = 0.0460$) (Table II). There was a statistically significant 26% difference in the complete and major control rates between the two arms.

In agreement with other investigators (Kris *et al.*, 1985*b*, 1989; Gandara *et al.*, 1992), the highest incidence of delayed emesis was observed on day 2 after cisplatin administration, and only three patients experienced first delayed emesis on day 4 or later in our study. Delayed emesis seems to become a smaller problem if it is well controlled on days 2 and 3. Therefore, every effort should be directed to obtaining perfect protection from both acute and delayed emesis on days 1–3.

Dose of cisplatin, control of acute emesis and gender have previously been reported as important determinants for delayed emesis (Kris *et al.*, 1985*a*; Roila *et al.*, 1991; Italian Group for Antiemetic Research 1994). In this study, dose of cisplatin ($80\,mg\,m^{-2}$ or $100\,mg\,m^{-2}$) was also not a significant factor. This may be because all patients received high-dose cisplatin (either dose level of $80\,mg\,m^{-2}$ or $100\,mg\,m^{-2}$) (Table II). Treatment arm, habitual high alcohol intake and control of acute emesis were significant variables in univariate analysis. Multivariate analysis using logistic regression models revealed that treatment arm (arm 1 or arm 2) was the only important factor ($P = 0.0101$) (Table III). A habitual high alcohol intake was the second, but did not reach statistical significance. The importance of complete control of acute emesis, which is often quoted as a significant determinant of subsequent antiemetic control, was not confirmed by multivariate analysis. This may be explained by different characteristics of population in this study, or by the relatively small number of patients, which provides only a low statistical power for detecting differences of moderate magnitude between the groups.

In conclusion, a combination of granisetron, dexamethasone and prochlorperazine was more effective than granisetron alone in protection from delayed emesis after high-dose cisplatin. Since 23% of patients treated with the combination regimen suffered from severe delayed emesis, further studies with other more effective combinations of antiemetics with different mechanisms of action are needed to improve results in this population.

**Acknowledgements**
This work was supported in part by a Grant-in-Aid for Cancer Research from the Japanese Ministry of Health and Welfare (5-42) and by a grant from Nippon Kayaku (Tokyo, Japan) and Smith-Kline Beecham (Tokyo, Japan).

### References

CHEVALLIER B ON BEHALF OF THE GRANISETRON STUDY GROUP. (1993). The control of acute cisplatin-induced emesis - a comparative study of granisetron and a combination regimen of high-dose metoclopramide and dexamethasone. *Br. J. Cancer*, **68**, 176–180.

CUNNINGHAM D, POPLE A, FORD HT, HAUTHORN J, GAZET J-C, CHALLONER T AND COOMBES RC. (1987). Prevention of emesis in patients receiving cytotoxic drugs by GR38032F, a selective 5-HT3 receptor antagonist. *Lancet*, **1**, 1461–1463.

GANDARA DR, HARVEY WH, MONAGHAN GG, PEREZ EA, STOKES C, BRYSON JC, FINN AL AND HESKETH PJ. (1992). The delayed-emesis syndrome from cisplatin: phase III evaluation of ondansetron versus placebo. *Semin. Oncol.*, **19**, (suppl. 10), 67–71.

GRALLA RJ, ITRI LM, PISKO SE, SQUILLANTE AE, KELSEN DP, BRAUN DW JR, BORDIN LA, BRAUN TJ AND YOUNG CW. (1981). Antiemetic efficacy of high-dose metoclopramide: randomized trials with placebo and prochlorperazine in patients with chemotherapy-induced nausea and vomiting. *N. Engl. J. Med.*, **305**, 905–909.

HAINSWORTH JD AND HESKETH PJ. (1992). Single-dose ondansetron for the prevention of cisplatin-induced emesis: efficacy results. *Semin. Oncol.*, **19**, (suppl. 15), 14–19.

HAMIK A AND PEROUTKA SJ. (1989). Differential interactions of traditional and novel antiemetics with dopamine $D_2$ and 5-hydroxytryptamine$_3$ receptors. *Cancer Chemother. Pharmacol.*, **24**, 307–310.

HERRSTEDT J, SIGSGAARD T, BOESGAARD M, JENSEN TP AND DOMBERNOUSKY P. (1993). Ondansetron plus metopimazine compared with ondansetron alone in patients receiving moderately emetogenic chemotherapy. *N. Engl. J. Med.*, **328**, 1076–1080.

ITALIAN GROUP FOR ANTIEMETIC RESEARCH. (1993). Difference in persistence of efficacy of two antiemetic regimens on acute emesis during cisplatin chemotherapy. *J. Clin. Oncol.*, **11**, 2396–2404.

ITALIAN GROUP FOR ANTIEMETIC RESEARCH. (1994). Cisplatin-induced delayed emesis: pattern and prognostic factors during three subsequent cycles. *Ann. Oncol.*, **5**, 585–589.

JANTUNEN IT, MUHONEN TT, KATAJA VV, FLANDER MK AND TEERENHOVI L. (1993). 5-HT3 receptor antagonists in the prophylaxis of acute vomiting induced by moderately emetogenic chemotherapy – a randomized study. *Eur. J. Cancer.*, **29A**, 1669–1672.

JOSS RA, DOTT CS. ON BEHALF OF THE GRANISETRON STUDY GROUP. (1993). Clinical studies with granisetron, a new 5-HT3 receptor antagonist for the treatment of cancer chemotherapy-induced emesis. *Eur. J. Cancer*, **29A**, (suppl. 1), S22–S29.

KAIZER L, WARR D, HOSKINS P, LATREILLE J, LOFTERS W, YAU J, PALMER M, ZEE B, LEVY M AND PATER J. (1994). Effect of schedule and maintenance on the antiemetic efficacy of ondansetron combined with dexamethasone in acute and delayed nausea and emesis in patients receiving moderately emetogenic chemotherapy: a phase III trial by the National Cancer Institute of Canada Clinical Trials Group. *J. Clin. Oncol.*, **12**, 1050–1057.

KAYE SB, KHAYAT D, AAPRO M AND DIEHL V. (1992). Who should receive a 5-HT-$_3$ antagonist? *Lancet*, **340**, 1107–1108.

KRIS MG, TYSON LB, GRALLA RJ, CLARK RA, ALLEN JC AND REILLY LK. (1983). Extrapyramidal reactions with high-dose metoclopramide. *N. Engl. J. Med.*, **309**, 433.

KRIS MG, GRALLA RJ, TYSON LB, CLARK RA, KELSEN DP, REILLY LK, GROSHEN S, BOSL GJ AND KALMAN LA. (1985*a*). Improved control of cisplatin-induced emesis with high-dose metoclopramide and with combinations of metoclopramide, dexamethasone, and diphenhydramine; results of consecutive trials in 255 patients. *Cancer*, **55**, 527–534.

KRIS MG, GRALLA RJ, CLARK RA, TYSON LB, O'CONNELL JP, WERTHEIM MS AND KELSEN DP. (1985*b*). Incidence, course and severity of delayed nausea and vomiting following the administration of high-dose cisplatin. *J. Clin. Oncol.*, **3**, 1379–1384.

KRIS MG, GRALLA RJ, TYSON LB, CLARK RA, CIRRINCIONE C AND GROSHEN S. (1989). Controlling delayed vomiting: double-blind, randomized trial comparing placebo, dexamethasone alone, and metoclopramide plus dexamethasone in patients receiving cisplatin. *J. Clin. Oncol.*, **7**, 108–114.

LOUVET C, LORANGE A, LETENDRE F, BEAULIEU R, PRETTY HM, COURCHESNE Y, NEEMEH JA, MONTE M AND LATREILLE J. (1991). Acute and delayed emesis after cisplatin-based regimen: description and prevention. *Oncology*, **48**, 392–396.

MORENO I, ROSELL R, ABAD A, BARNADAS A, CALRES J, RIBELLES N, SOLANO V AND FONT R. (1992). A comparison of three protracted antiemetic regimens for the control of delayed emesis in cisplatin-treated patients. *Eur. J. Cancer*, **28A**, 1344–1347.

ROILA F, TONATO M, COGNETTI F, CORTESI E, FAVALLI G, MARANGOLO M, AMADORI D, BELLA MA, GRAMAZIO V, DONATI D, BALLATORI E AND DEL FAVERO A. (1991). Prevention of cisplatin-induced emesis: a double-blind multicenter randomized crossover study comparing ondansetron and ondansetron plus dexamethasone. *J. Clin. Oncol.*, **9**, 675–678.

VON HOFF DD, SCHILSKY R, REICHERT CM, REDDICK RL, ROZENCWEIG M, YOUNG RC AND MUGGIA FM. (1979). Toxic effects of *cis*-dichlorodiammineplatinum(II) in man. *Cancer Treat. Rep.*, **63**, 1527–1531.

WORLD HEALTH ORGANIZATION. (1979). *WHO Handbook for Reporting Results of Cancer Treatment*. WHO Offset Publication No. 48. World Health Organization: Geneva.